# No significant long-term complications from inadvertent exposure to gonadotropin-releasing hormone agonist during early pregnancy in mothers and offspring: a retrospective analysis

**DOI:** 10.1186/s12958-021-00732-1

**Published:** 2021-03-20

**Authors:** Huan Wu, Xiaoyan Xu, Cong Ma, Yiran Zhou, Shanai Pei, Hao Geng, Ye He, Qianhua Xu, Yuping Xu, Xiaojin He, Ping Zhou, Zhaolian Wei, Xiaofeng Xu, Yunxia Cao

**Affiliations:** 1 Reproductive Medicine Center, Department of Obstetrics and Gynecology, The First Affiliated Hospital of Anhui Medical University, No. 218 Jixi Road, Hefei, 230022 China; 2 NHC Key Laboratory of Study on Abnormal Gametes and Reproductive Tract, Anhui Medical University, No. 81 Meishan Road, Hefei, 230032 China; 3 Key Laboratory of Population Health Across Life Cycle, Anhui Medical University, Ministry of Education of the People’s Republic of China, No. 81 Meishan Road, Hefei, 230032 China; 4 The Children’s Neurorehabilitation Center, Pediatric Department, The First Affiliated Hospital of Anhui Medical University, No. 218 Jixi Road, Hefei, 230022 China; 5 Anhui Province Key Laboratory of Reproductive Health and Genetics, No. 81 Meishan Road, Hefei, 230032 China; 6 Biopreservation and Artificial Organs, Anhui Provincial Engineering Research Center, Anhui Medical University, No. 81 Meishan Road, Hefei, 230032 China

**Keywords:** Gonadotropin-releasing hormone agonist, Neurodevelopment, Obstetric outcomes, Repregnancy, Teratogenicity

## Abstract

**Background:**

Administration of gonadotropin-releasing hormone agonist (GnRH-a) in the luteal phase is commonly used for pituitary suppression during in vitro fertilisation (IVF). There is an ineluctable risk of inadvertent exposure of spontaneous pregnancy to GnRH-a. However, little is known about the pregnancy complications and repregnancy outcomes of the affected women and the neurodevelopmental outcomes of the GnRH-a-exposed children.

**Methods:**

Retrospective analysis was used to determine obstetric and repregnancy outcomes after natural conception in 114 women who naturally conceived while receiving GnRH-a during their early pregnancy over the past 17 years. The GnRH-a-exposed children were evaluated to determine their neonatal characteristics and long-term neurodevelopmental outcomes. The outcomes were compared to those of relevant age-matched control groups.

**Results:**

Sixty-five women had 66 live births. The neonatal health outcomes and the incidence of maternal complications were similar in the GnRH-a-exposed and control groups. Thirty-one GnRH-a-exposed children, aged 2–8 years, were available for investigation of neurodevelopment. Except for one case of autism spectrum disorder, the full-scale intelligence quotient score was within the normal range and similar to that of the control group. Most mothers with successful pregnancies and about one-third of the women who had spontaneous abortions were subsequently able to conceive naturally again. IVF is recommended for repregnancy in women who have experienced ectopic pregnancies.

**Conclusions:**

Accidental exposure to GnRH-a in early pregnancy might be safe. Reproductive treatment suggestions for repregnancy should be made with consideration of the outcomes of the previously GnRH-a-exposed spontaneous pregnancy.

**Supplementary Information:**

The online version contains supplementary material available at 10.1186/s12958-021-00732-1.

## Background

Gonadotropin-releasing hormone agonist (GnRH-a) has been commonly used as a hypothalamic regulator for controlled ovarian stimulation (COS) in the cycle of in vitro fertilisation (IVF) [1]. The GnRH analogue is characterised by substitution of a D-amino acid for the glycine at position six of GnRH. This structural modification increases its affinity for the GnRH receptor and enhances its resistance to proteolytic degradation [2]. As one of the mainstream regimes for COS [1, 3, 4], the GnRH-a long protocol reduces the cancellation rates of COS, which is usually caused by premature luteinisation and spontaneous ovulation [5], and increases the number of retrieved oocytes as well as the clinical pregnancy rates [6, 7]. However, there is an ineluctable risk of inadvertent exposure of pregnancy to GnRH-a administered in the luteal phase of the conception cycle [8].

Several studies have reported no significantly abnormal outcomes of such spontaneous pregnancies that have involved accidental exposure to GnRH-a during COS [9–15]. Furthermore, some infertile patients could become pregnant repeatedly with the administration of GnRH-a [16–19]. These observations seem to indicate that the occurrence of these unexpected pregnancies is not merely coincidental, but that GnRH-a might play a positive role in promoting fertility. This hypothesis is strongly supported by the extrahypothalamic activities of GnRH, which is involved in reproductive processes at multiple levels. GnRH has been demonstrated to play a crucial role in improving the development of mammalian blastocysts [20–22], stimulating embryonic trophoblastic cell invasion [23, 24], inducing placental secretion of human chorionic gonadotropin (HCG) [25–27], and regulating the receptivity of the uterine endometrium to the embryo [28, 29]. Accumulating evidence of these advantages has led to the deliberate adoption of GnRH-a in some IVF cycles to promote embryo implantation, sometimes with positive results [30–32].

Although most of the individuals with GnRH-a-exposed pregnancy could give birth to healthy offspring, and the incidence of miscarriage and malformation in such cohorts was not significantly different from that in the general population [18], most follow-up studies were based only on early postnatal examination of the infants. Reports evaluating the long-term neurological outcomes of these affected children are quite rare [19, 33]. Therefore, a long-term follow-up survey is crucial for elucidating the potential impact of this GnRH analogue on foetation.

The current study retrospectively analysed the maternal complications and neonatal health outcomes of a large cohort of women in China who had experienced GnRH-a administration during early pregnancy over the past 17 years, and evaluated the long-term neurodevelopmental outcomes of their offspring. Furthermore, we investigated these women’s repregnancy outcomes. This long-term follow-up survey would provide insight into the effects of GnRH-a on early pregnancy and provide a basis for appropriate counselling of such patients on reproductive treatment.

## Methods

### Study population

Women who were inadvertently exposed to GnRH-a soon after conception in the Reproductive Medicine Center of the First Affiliated Hospital of Anhui Medical University from January 2003 to December 2019 were enrolled in this study. These couples had planned to undergo IVF due to infertility of various aetiologies, such as tubal damage, male factors, and recurrent spontaneous abortion (RSA). A long protocol of pituitary desensitisation was performed on days 21–23 of the previous cycle, either with a daily dose of 0.1 mg short-acting GnRH-a (Triptorelin, 0.1 mg; Ferring Pharma, Kiel, Germany) for 14 days, or with a single dose of 0.9–1.1 mg long-acting GnRH-a (Triptorelin, 3.75 mg; Beaufour Ipsen Pharma, Paris, France). The 3.75 mg triptorelin was mixed with 2 ml sterile water beforehand. The 0.48–0.59 ml solution containing 0.9–1.1 mg triptorelin was subsequently aspirated with a 1 ml sterile syringe for injection. The single dose of long-acting GnRH-a was adjusted according to the patients’ weight.

Couples were not advised to use contraceptive precautions during this downregulation phase but were informed that an inadvertent pregnancy could occur. Unexpected pregnancies were identified by routine assay of serum β-HCG on the 14th day after initiation of GnRH-a administration. Each couple chose to continue with the inadvertent pregnancy. Progesterone in oil was subsequently injected up to the 10th gestational week, at a dose of 40–60 mg per day. Clinical pregnancy was confirmed by transvaginal ultrasound 6 weeks after the occurrence of the last menses.

Available children who had been born to mothers who were exposed to GnRH-a during early pregnancy were also assessed through face-to-face interaction. In addition, two groups of age-matched children, born in our hospital, were included as control groups. One group contained 45 children who were born as the result of IVF, and the other group comprised 45 children who were born after spontaneous pregnancies.

All participants provided signed informed consent after an explanation of the study; furthermore, all parents of the children gave consent for their offspring to participate in the study. The research protocol was approved by the ethics committee of Anhui Medical University.

### Retrieval of medical records of obstetric characteristics and collection of repregnancy outcomes

Information regarding maternal complications, such as preterm delivery, gestational hypertension, gestational diabetes, placenta previa, and postpartum haemorrhage, was retrospectively analysed using the women’s hospital charts. We also reviewed the neonatal characteristics of the offspring, including mode of delivery, gestational age, birth weight, body height, Apgar score, and birth defects. Moreover, the repregnancy outcomes of these women were meticulously collected by telephonic follow-up.

### Investigation of the physical health and neurodevelopment of the affected children

The general physical health of all children was assessed by the same paediatrician. The Wechsler Preschool and Primary Scale of Intelligence—4th edition (WPPSI-IV, Chinese version) was used to investigate the neurodevelopment of all children aged 2–8 years. The Full-Scale Intelligent Quotient (FSIQ) score was calculated to represent the child’s intellectual abilities. Children with suspected impairments in social or executive functioning were more closely examined. Attention-deficit hyperactivity disorder (ADHD) and autism spectrum disorders (ASDs) were diagnosed based on the criteria of the Diagnostic and Statistical Manual of Mental Disorders—5th edition.

### Statistical analyses

Measurement data with a normal distribution are described using mean ± standard deviation, and count data are described using frequency and percentage. Student’s *t*-test was used to compare the means between two groups. Chi-square tests were used to compare proportions between the two groups. One-way analysis of variance was used to compare means among the three groups. *P*-values ≤0.05 indicated statistically significant differences. Statistical analyses were conducted using SPSS (Windows version 16.0, IBM-SPSS, Chicago, IL).

## Results

### Clinical data and pregnancy outcomes after inadvertent exposure to GnRH-a soon after conception

Among 26,002 IVF cycles conducted with GnRH-a long protocol at our centre over the past 17 years, 146 (0.56%) resulted in clinical pregnancy during the administration of GnRH-a. The number of annual GnRH-a long protocol-associated IVF cycles and the corresponding spontaneous conception cases involving exposure to GnRH-a are summarised in Fig. S1. Follow-up surveys were available for 114 (78.08%) mothers. Clinical data of the initial 146 couples, the 114 couples with follow-up available, and the 32 couples lost to follow-up are presented in Table S1.

**Fig. 1:**
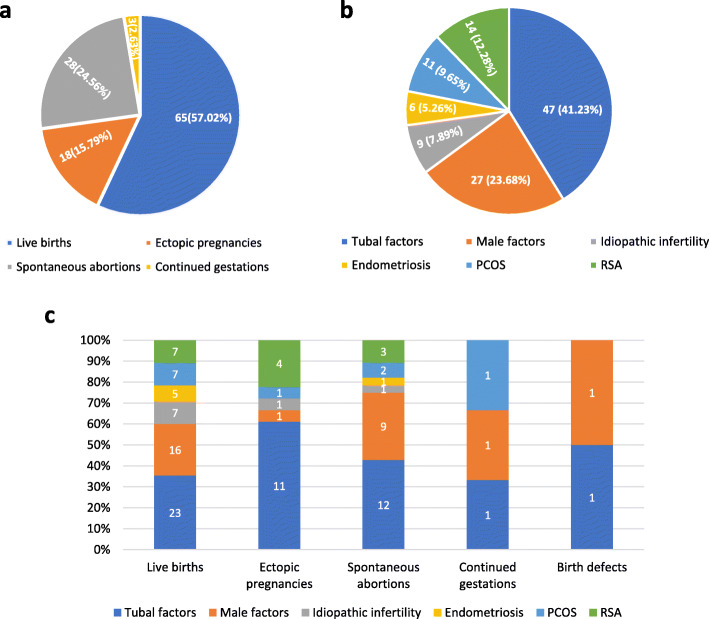
Observations on etiologic classification and pregnancy outcomes of the involved subjects. **a** For the 114 GnRH-a-related spontaneous pregnancies that could be followed up, 65 (57.02%) pregnancies resulted in live births, and another three (2.63%) pregnancies are ongoing. The remaining 46 (40.35%) pregnancies were lost as a result of ectopic pregnancy (15.79%) or spontaneous abortion (24.55%). **b** According to etiologic classification, the involved 114 subfertile couples consisted of 47 (41.23%) tubal damage cases, 27 (23.68%) sperm disorder cases, 14 (12.28%) recurrent spontaneous abortion (RSA) cases, 11 (9.65%) polycystic ovary syndrome (PCOS) cases, 6 (5.30%) endometriosis cases and 9 (7.89%) idiopathic infertile cases, respectively. **c** The subfertile cases with distinct etiologies were calculated separately based on the classification of pregnancy outcome. Tubal damage cases represented the main component in those groups. Furthermore, the proportion of tubal damage cases in ectopic pregnancies (61.11%) and the group of birth defects (66.67%) were higher than in other groups

Among the 114 women with inadvertent pregnancy, 65 (57.02%) gave birth to 66 children (including one twin pregnancy). Another three (2.63%) individuals were pregnant at the time of writing this manuscript. The remaining mothers (40.35%) had to terminate their pregnancies due to ectopic pregnancies (15.79%) or spontaneous abortion (24.55%) (Fig. 1a).

Tubal damage, male factors, and RSA accounted for the initial infertility in 47 (41.23%), 27 (23.68%), and 14 (12.28%) cases, respectively, and other causes were responsible for infertility in 26 (22.81%) cases (Fig. 1b). The composition of subfertile cases with distinct aetiology according to pregnancy outcome is summarised in Fig. 1c. There were no significant differences between the live birth group and the non-live birth group in terms of the baseline characteristics, such as couples’ age, duration of infertility, body mass index, basal sex hormone levels, number of antral follicles, and sperm parameters (Table 1).

**Table 1:** Clinical characteristics of the couples inadvertently exposed to GnRH-a soon after conception

Clinical parameters	Couples with unexpected pregnancies associated with GnRH-a (***n*** = 114)		***P*** value
	Women with live birth (***n*** = 65)	Women without live birth (***n*** = 49)	
Female general characteristics			
Age (y)	29.02 ± 3.42	30.06 ± 3.82	0.127
Duration of infertility (y)	3.13 ± 1.90	3.00 ± 2.49	0.749
Primary infertility cases (%)	30 (46.15)	26 (53.06)	0.465
BMI (kg/m^2^)	21.97 ± 3.20	21.04 ± 2.38	0.090
Basal serum sex hormone levels			
FSH (pmol/L)	6.94 ± 2.11	7.08 ± 1.75	0.711
LH (pmol/L)	5.30 ± 3.15	4.84 ± 2.26	0.726
E_2_ (pmol/L)	165.71 ± 133.16	173.99 ± 112.68	0.171
T (pmol/L)	1.41 ± 1.34	1.19 ± 0.46	0.380
PRL (pmol/L)	16.18 ± 11.53	19.31 ± 12.58	0.260
Number of antral follicles			
Right ovary	5.80 ± 2.29	5.33 ± 2.57	0.320
Left ovary	5.46 ± 2.46	5.78 ± 2.31	0.490
Male characteristics			
Age (y)	30.85 ± 4.68	31.69 ± 4.21	0.320
Sperm volume (ml)	3.52 ± 1.57	3.70 ± 1.59	0.542
Sperm concentration (10^6^/ml)	91.17 ± 81.43	70.15 ± 50.38	0.115
Progress motility (%)	44.43 ± 17.57	42.34 ± 15.08	0.247
Sperm abnormality rate (%)	90.33 ± 43.40	90.78 ± 43.85	0.433

Annotation: *BMI* Body mass index, *FSH* Follicle stimulating hormone, *LH* Luteinizing hormone, *E2* Estradiol, *T* Testosterone, *PRL* Prolactin

### Maternal complications of women who gave live birth after inadvertent exposure to GnRH-a

The rates of preterm delivery, gestational hypertension, and gestational diabetes among the 65 women who gave live birth after spontaneously conceiving with exposure to GnRH-a were 18.46, 4.62, and 4.62%, respectively. There were no instances of placenta previa or postpartum haemorrhage. Notably, the incidence of these complications in this cohort was comparable to that of the age-matched mothers who had conceived naturally or following IVF (Table S2).

### Neonatal characteristics and long-term neurodevelopmental outcomes of children born after exposure to GnRH-a

Neonatal health parameters, such as birth weight, body height, and Apgar score, of the 66 children who were born after exposure to GnRH-a were all within the normal range. Furthermore, except for one case of ventricular septal defect (VSD) and one case of cleft lip (CL), no other significant malformations occurred (Table S3). The VSD had closed naturally about 6 months after birth, and the CL had been surgically repaired by the age of 2 years.

Of the 66 children, only 31 (46.97%) children aged 2 to 8 years (5.03 ± 1.80) were available for investigation of neurodevelopment (Table 2). There was no evidence of any family history that might be associated with mental disorders in this cohort. Only one child was diagnosed with ASD. The general cognitive status was within the normal range in the other children, and their average FSIQ score was 105.77 ± 8.58.

**Table 2:** Neonatal characteristics and long-term neurodevelopment outcomes of the 31 children born following exposure to GnRH-a

Clinical data	Children born following exposure to GnRH-a (***n*** = 31)	Children born following IVF (***n*** = 45)	Children born following spontaneous conception (***n*** = 45)	***P*** value
Mode of delivery				
Caesarean section %^b^	40.0 (12/30)	64.44(29/45)	37.78(17/45)	0.023
Neonatal health outcomes				
Gestational weeks	37.97 ± 1.89	37.80 ± 2.37	38.60 ± 1.84	0.093
Singleton birth	29	33	45	–
Twins	1	6	0	–
Birth weight (g)	3175.81 ± 477.82	3160.00 ± 405.45	3308.89 ± 373.15	0.111
Body height (cm)	50.16 ± 2.53	49.73 ± 1.79	50.11 ± 1.19	0.449
Apgar score	9.97 ± 0.18	9.87 ± 0.34	9.93 ± 0.25	0.239
Birth defects	1 (Cleft lip)	1 (ASD)	0	–
Neurodevelopmental outcomes				
Children age (year)	5.03 ± 1.80	5.22 ± 1.99	5.16 ± 1.15	0.860
FSIQ^a^	105.77 ± 8.58	105.98 ± 10.61	104.60 ± 11.26	0.865
ADHD	0	2	1	–
ASDs	1	0	0	–

Annotation: ^a^ the child with ASDs was not evaluated; ^b^GnRH-a group vs IVF group (*p* = 0.037); *IVF* In vitro fertilisation, *ASD* Atrial septal defect, *FSIQ* Full-scale intelligence quotient, *ADHD* Attention-deficit hyperactivity disorder, *ASDs* Autism spectrum disorders

In addition, the neonatal health and long-term neurodevelopmental outcomes of the 31 children with GnRH-a exposure were compared with those of 45 IVF-conceived children and 45 naturally conceived children. One child in the IVF group was diagnosed with an atrial septal defect. The caesarean section rate of the study group was significantly lower than that of the IVF group (*p* = 0.037). There were no statistically significant differences between the study group and the two control groups in terms of gestational weeks, birth weight, body height, and Apgar scores. In terms of neurodevelopmental outcomes, two subjects in the IVF group and one in the spontaneous conception group had ADHD features. The average FSIQ score of the study group were comparable to those of the control groups.

### Repregnancy outcomes of the 62 women with GnRH-a-exposed spontaneous pregnancies

**Fig. 2:**
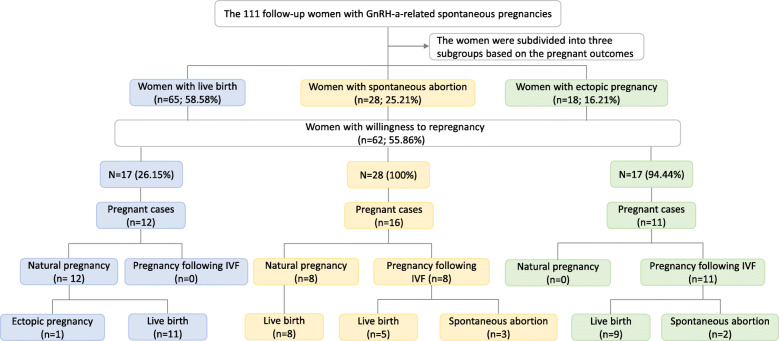
Repregnancy outcomes of the women with discrepant results of the GnRH-a-related spontaneous pregnancies. 62 women with the willingness to repregnancy were subdivided into three subgroups based on outcomes of the previous GnRH-a-related spontaneous pregnancy. Most (70.59%) of the women who had given birth successfully were still able to conceive naturally and give birth successfully. 16 (57.14%) women who had experienced spontaneous abortion during the unexpected pregnancy got re-pregnant, half naturally and half following IVF, resulting in 13 live births and three spontaneous abortions. 11 (64.71%) women who had suffered from ectopic pregnancy got re-pregnant following IVF, resulting in nine live births and two spontaneous abortions

The repregnancy outcomes of the 62 (55.86%) women who wanted an additional child or desired to become pregnant again after a spontaneous abortion or ectopic pregnancy are summarised in Fig. 2. The individuals were subdivided into three subgroups based on the outcomes of the previous GnRH-a-related spontaneous pregnancy. For the 65 women who had given birth successfully, 17 (26.15%) desired to have a second child, and 12 spontaneous pregnancies culminated in 11 live births and 1 ectopic pregnancy. All 28 women with previous spontaneous abortions desired to become pregnant again, and 16 succeeded: eight conceived naturally and gave birth, while the other eight became pregnant after IVF, and gave live birth (five) or suffered spontaneous abortion (three). The remaining 17 (94.44%) women who had experienced ectopic pregnancies chose IVF to attempt reproduction. Ultimately, nine of them gave live birth and two suffered spontaneous abortion.

## Discussion

We investigated the obstetric and repregnancy outcomes in women who inadvertently conceived naturally after being administered GnRH-a during the course of IVF treatments and the neurodevelopmental outcomes of the GnRH-a-exposed children. More than half of the participants gave live birth. The neonatal health outcomes and the incidence of maternal complications in the GnRH-a-exposed group were comparable to those of the control groups. Except for one case of ASD, the full-scale intelligence quotient score of the offspring was within the normal range and similar to that of the control group. Most mothers with successful pregnancies and about one-third of the women who had spontaneous abortion were subsequently able to naturally conceive again.

Given that GnRH-a is administered in the mid-luteal phase of the previous cycle to suppress pituitary function during long protocol COS, spontaneous pregnancies with accidental exposure to GnRH-a are unavoidable; patients may be reluctant to adopt contraceptive measures during the downregulation period, or may even have been recommended to attempt pregnancy during this period by some reproductive centres. Although GnRH has multiple extrahypothalamic functions that promote the development and implantation of mammalian embryos, it is possible that temporary alleviation of the couples’ infertility-specific anxiety during the pituitary downregulation cycle might assist pregnancy [34].

The positive prognosis of spontaneous pregnancies after exposure to GnRH-a at a very early stage has been well substantiated by numerous published reports, accounting for approximately 1% of long protocol IVF cycles [9, 16]. This report of 114 cases represents a sizable number of pregnancies involving exposure to GnRH-a and demonstrates the long-term physical as well as neurological outcomes of the related offspring. The incidence of these unexpected pregnancies per cycle was 0.56% at our centre. For the 114 traceable cases, tubal damage and sperm disorder represented the main causes of infertility, similar to other reports [9, 14], indicating subfertility rather than absolute infertility in these patients. Intramuscular progesterone was routinely applied to correct the probable luteal phase defect caused by the luteolytic properties of GnRH-a [35]; however, the pregnancy loss rate (40.34%), including ectopic pregnancies and spontaneous abortions, in our cohort was higher than that of previous studies (around 25%) [9]. This may be related to the 32 cases in which patients were lost to follow-up. The absence of additional assisted reproductive treatment records for these excluded couples at our centre suggests that these individuals with unexpected pregnancies might have succeeded in having offspring. With these putatively successful cases, the high pregnancy loss rates would be reduced to more typical levels.

Teratogenicity always needs to be considered in these circumstances. In our retrospective analysis of the perinatal characteristics of this large cohort, we confirmed the healthy outcomes of children after inadvertent GnRH-a exposure during early pregnancy. No fatal congenital deformities occurred in any of the 66 children assessed. Two cases of common malformations, including VSD and CL, were identified. These common birth defects are associated with multifactorial origins, such as environmental factors, genetic defects, and maternal features [36]. VSD is the commonest congenital heart defect, with an incidence of approximately 3/1000 in Chinese neonates [37], and spontaneous closure occurs during childhood in most cases [38]. The prevalence rate of CL is around 1/1000 in China [39]. This common malformation is typically effectively repaired by plastic surgery. A large retrospective study in Australia reported two non-fatal malformations, bilateral inguinal hernia and unilateral kidney atresia, in a cohort of 42 children born to mothers with GnRH-a exposure [18]. In addition, the neonatal characteristics of our study group in terms of birth weight, body height, and Apgar score were comparable to those of the IVF and spontaneous delivery groups. Although these favourable neonatal health outcomes could theoretically be explained by the timing of exposure to GnRH-a (early pregnancy, a period before organogenesis), further investigation based on a larger cohort is required to elucidate the potential influence of early GnRH-a exposure on foetation outcomes.

Among the various obstetric conditions known to increase the risk of maternal morbidity, multiple pregnancies and advanced maternal age are universally recognised factors. These confer higher risk of pathological changes with severe effects on the mother [40, 41]. In our study cohort, no increase in obstetric complications of any sort was observed. It is tempting to speculate that short-term administration of GnRH-a in the early stage of pregnancy may not have any effect on maternal complications. Nevertheless, the young age of the couples and the prevalence of singleton pregnancies in the study group may have influenced the favourable obstetric outcomes. Furthermore, the sample size in the current study was insufficient for drawing definite conclusions.

Long-term neurodevelopment data on GnRH-a-exposed children is scarce. Lahat et al. [33] reported the long-term neurodevelopment outcomes of six GnRH-a-exposed children aged 3–9 years. Four of the six physically healthy children were diagnosed with ADHD, which could have been missed at an earlier assessment. This finding emphasises the importance of a long-term follow-up survey for these affected children. We investigated the intelligence and neurological development of 31 children who were inadvertently exposed to GnRH-a, and only one case of ASD was identified. For the other children, the general cognitive status was within the normal range, and their FSIQ scores were similar to those of the IVF-conceived and naturally conceived children. ASD, a neurological disorder characterised by impaired social communication and stereotypic behaviour, occurs in about 1% of the population. Genetic, epigenetic, and environmental factors are hypothesised to be associated with this disorder [42]. Interestingly, we found three cases of ADHD in the control groups. ADHD is another neurological disorder that also typically has an onset in childhood, with a prevalence of 3% [43]. These observations highlight the complexity of the aetiologies of these common neurodevelopmental disorders, suggesting the potentially causative association with obstetric factors, rather than with GnRH-a per se. Although the current results further reaffirm the previous proposal that accidental GnRH-a exposure in early pregnancy might be safe [18], a worldwide central registry of GnRH-a exposed pregnancies is urgently needed.

Guidelines for repregnancy in women with GnRH-a exposed pregnancy are currently lacking due to the absence of reliable evidence. We investigated the repregnancy outcomes of the women who had had various outcomes after their previous unexpected pregnancies. Most of the mothers who were desirous of repregnancy were able to conceive naturally and give birth successfully. Nearly half of the individuals who experienced spontaneous abortion during the unexpected pregnancy subsequently gave live birth. Notably, most of these pregnancies were established naturally. In cases with ectopic pregnancy, the couples clearly preferred IVF for repregnancy, and favourable outcomes were consistently achieved with this technique. Our retrospective analysis provides a basis for appropriate counselling and reproductive treatment suggestions for repregnancy in the affected couples.

## Conclusions

There is currently no indication of adverse effects subsequent to inadvertent GnRH-a exposure during the early pregnancy period. Such results have been reasonably consistent across geographical regions. Whether these consistent observations justify the recommendation for avoiding contraception during GnRH-a-related pituitary downregulation or utilisation of GnRH-a for promoting IVF success during embryo transfer is not completely clear and requires further specific studies. The reproductive treatment suggestions for repregnancy in women who have become pregnant during GnRH-a administration should take into consideration the specific outcomes of the previous unexpected pregnancy.

## Supplementary Information

**Additional file 1: Table S1.** Clinical characteristics of the enrolled couples exposed to GnRH-a soon after conception. **Table S2.** Maternal complications of the women with live birth inadvertently exposed to GnRH-a. **Table S3.** Neonatal characteristics of the 66 children born after exposure to GnRH-a. **Figure S1.** The number of annual GnRH-a long protocol-associated IVF cycles and the corresponding spontaneously conceived cases from 2003 to 2019.

## Data Availability

The datasets used and/or analysed during the current study are available from the corresponding author on reasonable request.

## Abbreviations

GnRH-a: Gonadotropin-releasing hormone agonist
COS: Controlled ovarian stimulation
IVF: In vitro fertilisation
HCG: Human chorionic gonadotropin
RSA: Recurrent spontaneous abortion
FSIQ: Full-Scale Intelligent Quotient
ADHD: Attention-deficit hyperactivity disorder
VSD: Ventricular septal defect
CL: Cleft lip
