# Supplementary material for: No significant long-term complications from inadvertent exposure to gonadotropin-releasing hormone agonist during early pregnancy in mothers and offspring: a retrospective analysis
Source: Reprod Biol Endocrinol. 2021 Mar 20;19:46. doi: 10.1186/s12958-021-00732-1 (PMC7980339; doi:10.1186/s12958-021-00732-1)
Supplement: Supplementary file 1 — Additional file 1: Table S1. Clinical characteristics of the enrolled couples exposed to GnRH-a soon after conception. Table S2. Maternal complications of the women with live birth inadvertently exposed to GnRH-a. Table S3. Neonatal characteristics of the 66 children born after exposure to GnRH-a. Figure S1. The number of annual GnRH-a long protocol-associated IVF cycles and the corresponding spontaneously conceived cases from 2003 to 2019. [file 12958_2021_732_MOESM1_ESM.pdf]

Supplementary materials for 'No significant long-term complications from inadvertent exposure to gonadotropin-releasing hormone agonist during early pregnancy in mothers and offspring: a retrospective analysis'

Contents

Table S1. Clinical characteristics of the enrolled couples exposed to GnRH-a soon after conception

Table S2. Maternal complications of the women with live birth inadvertently exposed to GnRH-a

Table S3. Neonatal characteristics of the 66 children born after exposure to GnRH-a

Figure S1. The number of annual GnRH-a long protocol-associated IVF cycles and the corresponding spontaneously conceived cases from 2003 to 2019

Table S1. Clinical characteristics of the enrolled couples exposed to GnRH-a soon after conception

Clinical parameters	Women with unexpected pregnancy (n=146)	Women with follow-up (n=114)	Women lost to follow-up (n=32)
Proportion in total 34457 cycles (%)	0.42	0.33	0.09
Female general characteristics			
Women age (y)	29.61±3.72	29.46±3.61	30.13±4.08
Duration of infertility (y)	2.98±2.16	3.08±2.16	2.62±2.16
Primary infertility cases (%)	76(52.05)	56(49.12)	20(62.50)
BMI (kg/m^2)	21.83±3.36	21.57±2.90	22.75±4.57
Basal serum sex hormone levels			
FSH (pmol/L)	7.02±2.06	7.00±1.95	7.08±2.42
LH (pmol/L)	5.08±3.28	5.10±2.80	5.01±4.66
E2 (pmol/L)	170.32±132.22	169.27±124.31	174.06±159.44
T (pmol/L)	1.45±1.56	1.32±1.06	1.91±2.64
PRL (pmol/L)	17.06±11.39	17.53±12.04	15.38±8.64
No. of sinus follicles			
Right ovary	5.62±2.41	5.60±2.42	5.69±2.44
Left ovary	5.53±2.31	5.60±2.39	5.28±1.99
Male characteristics			
Men age (y)	31.44±4.61	31.21±4.48	32.25±5.04
Sperm volume (ml)	3.73±1.61	3.59±1.57	4.23±1.64
Sperm concentration (10^6/ml)	78.50±73.85	82.06±70.19	65.92±85.59
Progress motility (%)	42.63±16.30	43.51±16.47	39.59±15.70

Annotation: BMI, body mass index; FSH, follicle stimulating hormone; LH, luteinizing hormone; E2, estradiol; T, testosterone; PRL: prolactin.

Table S2. Maternal complications of the women with live birth inadvertently exposed to GnRH-a

Clinical data	Women with live birth inadvertently exposed to GnRH-a (n=65)	Women conceived following IVF (n=100)	Women conceived naturally (n=100)	*p* value
Baseline parameters				
Women age (y)	29.02±3.42	29.35±3.47	29.41±3.56	0.769
Maternal complications				
Preterm birth	12 (18.46%)	16 (16.0%)	10 (10.0%)	0.265
Gestational hypertension	3 (4.62%)	6 (6.0%)	3 (3.0%)	0.586
Gestational diabetes	3 (4.62%)	5(5.0%)	6 (6.0%)	0.916
Placenta previa	0	2 (2.0%)	1 (1.0%)	0.360
Postpartum hemorrhage	0	0	1 (1.0%)	0.376

Table S3. Neonatal characteristics of the 66 children born after exposure to GnRH-a

Clinical data	66 GnRH-a exposed Children	
	**Mean ± standard deviation**	**Min-max**
Current age (year)	4.80±3.64	0.5-16
Mode of delivery		
Vaginal %	59.09 (39/66)	-
Caesarian section %	40.91 (27/66)	-
Neonatal health outcomes		
Gestational weeks	38.23±1.75	34-41
Singleton birth	65	-
Twins	1	-
Birth weight (g)	3283.08±461.59	2100-4300
Body height (cm)	50.14±1.81	44-53
Apgar score	9.86±0.46	8-10
Birth defects		
Cleft lip	1	-
VSD	1	-

Annotation: VSD, ventricular septal defect.

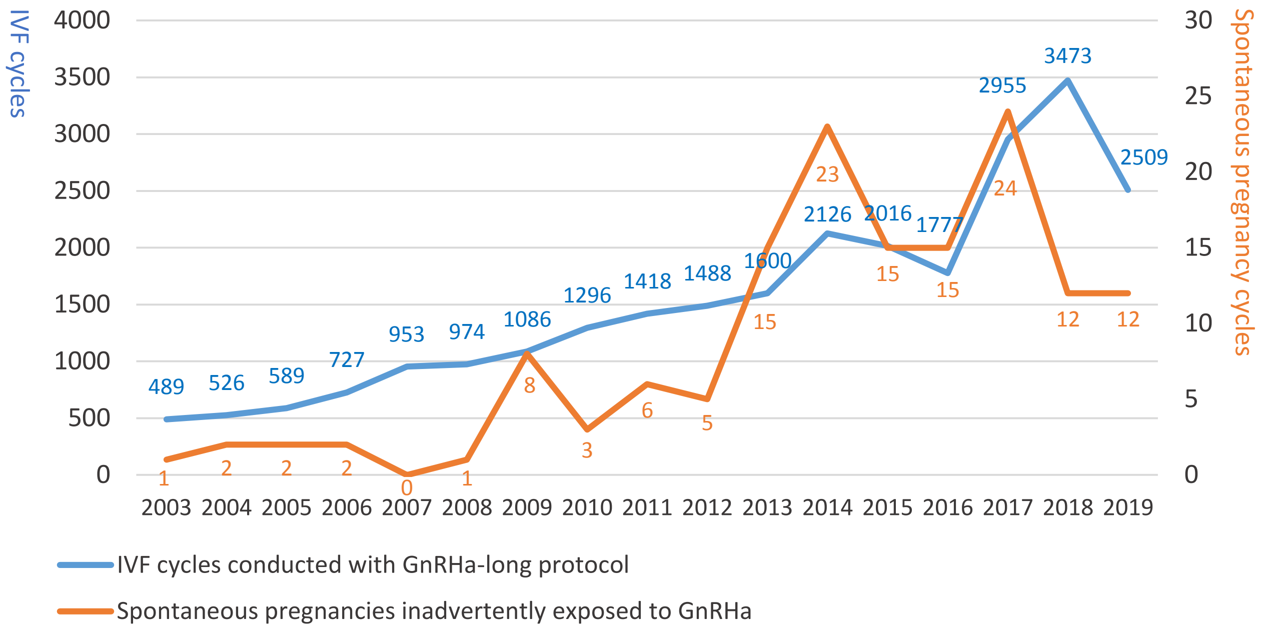

Figure S1. The number of annual GnRH-a long protocol-associated IVF cycles and the corresponding naturally conceived cases which were inadvertently exposed to GnRH-a during the down regulation period from 2003 to 2019 in our reproductive center. Out of the total 26,002 IVF cycles, 146 (0.56%) cycles had got clinical pregnancy during the commenced administration of GnRH-a. The average annual GnRH-a-related spontaneous pregnancy was 8.59±7.75.
